# ﻿The systematic position of puzzling Sino-Himalayan *Lophocoleasikkimensis* (Lophocoleaceae, Marchantiophyta) is identified

**DOI:** 10.3897/phytokeys.206.84227

**Published:** 2022-08-25

**Authors:** Vadim A. Bakalin, Yulia D. Maltseva, Ksenia G. Klimova, Van Sinh Nguyen, Seung Se Choi, Aleksey V. Troitsky

**Affiliations:** 1 Botanical Garden-Institute FEB RAS, Makovskogo Street 142, Vladivostok 690024, Russia Botanical Garden-Institute FEB RAS Vladivostok Russia; 2 Institute of Ecology and Biological Resources, Graduate University of Science and Technology, Vietnam Academy of Science and Technology, 18 Hoang Quoc Viet, Cau Giay, Ha Noi 10000, Vietnam Graduate University of Science and Technology Ha Noi Vietnam; 3 Team of National Ecosystem Survey, National Institute of Ecology, Seocheon 33657, Republic of Korea Team of National Ecosystem Survey, National Institute of Ecology Seocheon Republic of Korea; 4 Belozersky Institute of Physico-Chemical Biology, Lomonosov Moscow State University, Leninskie Gory 1, Moscow 119991, Russia Lomonosov Moscow State University Moscow Russia

**Keywords:** *Lophocolea*, Lophocoleaceae, molecular phylogenetic, Sino-Himalaya, taxonomy, Vietnam

## Abstract

*Lophocoleasikkimensis*, a little-known Sino-Himalayan species, was collected in North Vietnam and its taxonomic position was identified by molecular genetic techniques. The species is characterized by generally narrowly pointed leaves, which are not seen in other representatives of Lophocoleaceae. We found that it belongs to the recently described genus *Cryptolophocolea*, although it is clearly morphologically dissimilar to other members of the genus. We propose a corresponding nomenclature combination: *Cryptolophocoleasikkimensis* comb. nov. This species is the only one in its genus with a predominantly Sino-Himalayan distribution; the vast majority of congeners are distributed in the Southern Hemisphere (mostly in Australasia). Reports of this species in Vietnam further confirm the close phytogeographic relationships of the flora of northern Indochina with those of the Sino-Himalayas and suggest that this species is found in other parts of the Hoang Lien Range and the southern Hengduan Range.

## ﻿Introduction

*Lophocoleasikkimensis* (Steph.) Herzog & Grolle is a poorly known Lophocoleaceae species but is so different from other known members of the family that it appears to belong to a different genus. Kitagawa (1974) discussed similar considerations based on the first impressions of this taxon (l.c.: 32). The specific features (mostly not unique to *Lophocolea* s.l.) include ovate leaves with acute to obtuse apices, almost rounded underleaves distinctly connate to leaves on both sides, and biseriate antheridium stalk. Kitagawa (1974) described the history of the taxonomic interpretation of the species in detail, eventually concluding that the placement of the species in *Lophocolea* was appropriate. This point of view was adopted by Söderström et al. (2016). After we collected this species in Vietnam (Bakalin et al. 2018), we were impressed by its unusual appearance and decided to review the taxonomic position of this species, including molecular genetic methods that were, of course, unavailable to N. Kitagawa fifty years ago. This attempt seemed particularly appropriate in light of recent perturbations of Lophocoleaceae systematic over the last 10–15 years, which are clearly described by Söderström et al. (2013: 36): “Lophocoleaceae is a family with a turbulent history and many taxa have been moved back and forth among genera”. Thus, the goal of this work was to determine the taxonomic position of the species known as *Lophocoleasikkimensis*.

## ﻿Historical background

Initially, *Lophocoleasikkimensis* was placed in the genus *Herpocladium* as *H.sikkimense* (Stephani 1922); this genus contained heterogeneous elements (at least 4 genera) and is synonymous with *Herbertus* by its type species. Since the species was “hidden” within a contradictory treated genus, the same taxon was independently described 17 years later as *Lophocoleatrollii* by Herzog (1939). This name was synonymized with *Herpocladiumsikkimense* until the species was transferred to *Lophocolea* 20 years later (Herzog and Grolle 1958).

After reviewing the characteristics previously mentioned (plant color, large entire underleaves, dorsally secund leaves, biseriate antheridium stalk, etc.), Kitagawa (1974) concluded that some were not unique to that genus (as it was treated in that time) or were subject to variability within the species. After this comprehensive work, no additional special taxonomic investigations of this species were conducted. However, the generic position of this species was changed due to the broad genus concept of the Lophocoleaceae family (or that of the broadly defined Geocalycaceae family). Engel and Schuster (1985) proposed a broad concept: the *Chiloscyphus-Lophocolea* complex that fused both genera under an older name (*Chiloscyphus*), and therefore renamed the species in question as *Chiloscyphussikkimensis* (Steph.) J.J. Engel & R.M. Schust. This concept was received somewhat critically and was only limitedly accepted.

In the mid-2000s, important molecular-genetic comparisons were carried out on Lophocoleaceae, providing a new perspective on the old problem. Research by Hentschel et al. (2006a, 2006b, 2007) made it clear that Engel and Schuster (1985) were correct: it is impossible to distinguish only two genera in the *Chiloscyphus-Lophocolea* complex because the second genus then becomes clearly polyphyletic. The solution may be to either unite both genera (as done by Engel and Schuster 1985) or to divide *Lophocolea* into several genera. Hentschel et al. pursued the latter method. The most important entity segregated based on the molecular-genetic approach was *Cryptolophocolea*, which was described in 2013 (Söderström et al. 2013). This genus is characterized by a number of features, some of which (bifid, opposite to subopposite leaves, 2–4(–6)-lobed underleaves) are not observed in *Lophocoleasikkimensis*; other features of this genus, such as the biseriate antheridial stalk, large underleaves connate with leaves at both sides, indicate a possible relationship between it and *Lophocoleasikkimensis*.

## ﻿Methods

### ﻿Taxon sampling

We analyzed two specimens of *Lophocoleasikkimensis* in our molecular phylogenetic study using nucleotide sequence data from ribosomal operon of nuclear DNA (ITS1–2) and *trn*L-F of chloroplast DNA. In addition to Lophocoleaceae, the analysis included sequence data from genetically related Jungermanniales families (Cooper et al. 2011; Patzak et al. 2016). The outgroups in the ITS1-2-based tree were Herbertaceae species (*Herbertusdicranus* (Gottsche, Lindenb. & Nees) Trevis., *Triandrophyllumsubtriﬁdum* (Hook.f. & Taylor) Fulford & Hatcher), and Lepicoleaceae species (*Lepicoleaattenuata* (Mitt.) Steph., *Lepicoleascolopendra* (Hook.) Dumort. ex Trevis., *Lepicoleaochroleuca* (Spreng.) Spruce) for the *trn*L-F tree that is correspond to the topologies in Patzak et al. (2016). All sequences except those newly obtained were downloaded from GenBank.

There were too few *trn*G-intron sequences from Lophocoleaceae in GenBank to construct a reliable phylogenetic tree for this marker that establishes the position of *Lophocoleasikkimensis*.

Therefore, new *trn*G-intron sequences were obtained for this taxon but not analyzed properly.

Specimen voucher details, as well as newly identified and previously identified sequences, are listed in Table 1.

**Table 1.** The list of voucher details and GenBank accession numbers for the specimens used in phylogenetic reconstructions in the present paper (* *trn*G-intron GenBank accession number: OK562104; ** *trn*G-intron GenBank accession number: OK562105). Newly obtained sequences are in bold.

Initial species name	Accepted name	Label	GenBank accession number	
			ITS1–2	*trn*L-F
*Bragginsellaanomala* R.M. Schust.	*Bragginsellaanomala* R.M. Schust.	New Zealand, M. von Konrat & J.J. Engel, L1129 (F)	–	KJ802081
*Chiloscyphusaustrigenus* (Hook. f. & Taylor) J.J. Engel & R.M. Schust.	*Pachyglossaaustrigena* (Hook. f. & Taylor) L. Söderstr.	Chile, Hyvönen et al. 5793 (JE)	AM282805	–
*Chiloscyphusciliolatus* (Nees) J.J. Engel & R.M. Schust.	*Cryptolophocoleaciliolata* (Nees) L. Söderstr., Crand.-Stotl., Stotler & Váňa	Indonesia, Gradstein, 10327 (GOET)	AM491286	–
*Chiloscyphusconnatus* (Sw.) J.J. Engel & R.M. Schust.	*Cryptolophocoleaconnata* (Sw.) L. Söderstr. & Váňa	Costa Rica, Gradstein, 9404 (GOET)	AM282806	–
*Chiloscyphuscostatus* (Nees) J.J.Engel & R.M.Schust.	*Cryptolophocoleacostata* (Nees) L. Söderstr.	Malaysia, Schäfer-Verwimp & Verwimp, 18724/A (JE)	AM282807	–
*Chiloscyphuscucullistipulus* (Steph.) Hässel	*Clasmatocoleacucullistipula* (Steph.) Grolle	Chile, Drehwald, 970184 (GOET)	AM491287	–
*Chiloscyphuscuspidatus* (Nees) J.J. Engel & R.M. Schust.	*Lophocoleacuspidata* (Nees) Limpr.	Germany, Hentschel, Bryo 01411 (JE)	AM491604	–
*Chiloscyphusfragmentissimus* (R.M.Schust.) J.J.Engel & R.M.Schust.	*Lophocoleafragmentissima* R.M. Schust.	Venezuela, Frahm, 97/5/N (GOET)	AM282809	–
*Chiloscyphusfragrans* (Moris & De Not.) J.J.Engel & R.M.Schust.	*Lophocoleafragrans* (Moris & De Not.) Gottsche, Lindenb. & Nees	Azores, Schwab, 113 (JE)	AM282810	–
*Chiloscyphusfulvellus* (Hooker f. & Taylor) Nees	*Clasmatocoleafulvella* (Hook. f. & Taylor) Grolle	Chile, Hyvönen, 5313 (GOET)	AM491288	–
*Chiloscyphusgayanus* (Mont.) Gottsche & al.	*Clasmatocoleagayana* (Mont.) Grolle	Chile, Holz & Franzaring, CH 00-151a (GOET)	AM491289	–
*Chiloscyphusgottscheoides* (Besch. & C.Massal.) J.J.Engel & R.M.Schust.	*Pachyglossagottscheoides* (Besch. & C. Massal.) L. Söderstr.	Chile, Drehwald & Mues, 3239 (GOET)	AM282811	–
*Chiloscyphusguadalupensis* (Steph.) J.J.Engel & R.M.Schust.	*Cryptolophocoleaguadalupensis* (Steph.) L. Söderstr. & Váňa	Costa Rica, Gradstein & Mues, 9630 (GOET)	AM282813	–
*Chiloscyphushelmsianus* (Steph.) J.J. Engel & R.M. Schust.	*Cryptolophocoleahelmsiana* (Steph.) L. Söderstr.	New Zealand, Engel & von Konrat, 28439	–	FJ173297
*Chiloscyphushumilis* (Hook. f. & Taylor) Hässel	*Clasmatocoleahumilis* (Hook.f. et Taylor) Grolle	Chile, Holz & Franzaring, CH 00-44B (GOET)	AM491290	–
*Chiloscyphusitoanus* (Inoue) J.J. Engel & R.M. Schust.	*Lophocoleaitoana* Inoue	China, 1999 Piippo, 60709	–	AY149868
*Chiloscyphusjaponicus* (Steph.) J.J. Engel & R.M. Schust.	*Cryptolophocoleacompacta* (Mitt.) L. Söderstr.	China, 1998 Koponen et al. 50238	–	AY149869
*Chiloscyphuslentus* (Hook.f. & Taylor) J.J.Engel & R.M.Schust.	*Lophocolealenta* (Hook. f. & Taylor) Gottsche, Lindenb. & Nees	New Zealand, Engel & von Konrat, 24002	–	FJ173298
*Chiloscyphusleucophyllus* (Hook.f. & Taylor) Gottsche, Lindenb. & Nees	*Cryptolophocolealeucophylla* (Hook. f. & Taylor) L. Söderstr.	New Zealand, Engel & von Konrat, 24319	–	FJ173299
*Chiloscyphusliebmannianus* (Gottsche) J.J.Engel & R.M.Schust.	*Lophocolealiebmanniana* Gottsche	Mexico, Burghardt Bryo, 01655 (GOET)	AM282816	–
*Chiloscyphusmartianus* (Nees) J.J.Engel & R.M.Schust.	*Cryptolophocoleamartiana* (Nees) L. Söderstr., Crand.-Stotl. & Stotler	Ecuador, Gradstein, 10119 (GOET)	AM282817	–
*Chiloscyphusmassalongoanus* Steph.	*Cryptolophocoleamassalongoana* (Schiffn.) L. Söderstr.	Indonesia, Schaefer-Verwimp & Verwimp, S-V 25279	AM491292	–
*Chiloscyphusminor* (Nees) J.J.Engel & R.M.Schust.	*Lophocoleaminor* Nees	Germany, Hentschel Bryo, 01006 (JE)	AM282818	–
*Chiloscyphusminor* (Nees) J.J.Engel & R.M.Schust.	*Lophocoleaminor* Nees	China, Hunan Province, Zhangjiajie, 1999 Rao, 58428	–	AY149864
*Chiloscyphusmuricatus* (Lehm.) J.J.Engel & R.M.Schust.	*Lophocoleamuricata* (Lehm.) Nees	Australia, Streimann, 51629 (JE)	AM282819	–
*Chiloscyphusnovae-zeelandiae* (Lehm. & Lindenb.) J.J.Engel & R.M.Schust.	*Lophocoleanovae-zeelandiae* (Lehm. & Lindenb.) Nees	Australia, Eggers, AUS 3/81 (JE)	AM282820	–
Chiloscyphusnovae-zeelandiaevar.grandistipulus (Schiffn.) J.J.Engel	Lophocoleanovae-zeelandiaevar.grandistipula (Schiffn.) Váňa	New Zealand, Engel & von Konrat, 24120	–	FJ173300
*Chiloscyphusobvolutus* (Hook. f. & Taylor) Hässel	*Clasmatocoleaobvoluta* (Hook. f. & Taylor) Grolle	Chile, Hyvoenen, 2827 (GOET)	AM491293	–
*Chiloscyphuspallescens* (Hoﬀm.) Dumort.	*Chiloscyphuspallescens* (Hoﬀm.) Dumort.	Germany, Thuringia, Hentschel Bryo, 01418 (JE)	AM282821	–
*Chiloscyphuspallescens* (Hoﬀm.) Dumort.	*Chiloscyphuspallescens* (Hoﬀm.) Dumort.	Bulgaria, Hentschel Bryo, 0772 (JE)	AM282825	–
*Chiloscyphuspallescens* (Hoﬀm.) Dumort.	*Chiloscyphuspallescens* (Hoﬀm.) Dumort.	Poland, 1993 A. Stenel (W-4)	–	AY149871
*Chiloscyphusperissodontus* (Spruce) J.J.Engel & R.M.Schust.	*Cryptolophocoleaperissodonta* (Spruce) L. Söderstr.	Guyana, Gradstein, 4890 (GOET)	AM282826	–
*Chiloscyphusperissodontus* (Spruce) J.J.Engel & R.M.Schust.	*Cryptolophocoleaperissodonta* (Spruce) L. Söderstr.	Guyana, Gradstein, 5042 (GOET)	AM282827	–
*Chiloscyphusplatensis* J.J. Engel & R.M. Schust.	*Lophocoleaplatensis* C. Massal.	Bolivia, Churchill et. al., 20950 (JE)	AM491295	–
*Chiloscyphusplatensis* J.J. Engel & R.M. Schust.	*Lophocoleaplatensis* C. Massal.	Bolivia, Churchill et. al., 22090 (GOET)	AM491294	–
*Chiloscyphuspolyanthos* (L.) Corda	*Chiloscyphuspolyanthos* (L.) Corda	Slovakia, Hentschel Bryo, 0318 (JE)	AM282831	–
*Chiloscyphuspolyanthos* (L.) Corda	*Chiloscyphuspolyanthos* (L.) Corda	Finland, 2000 He-Nygren & Piippo, 1469	–	AY149873
*Chiloscyphuspolychaetus* (Spruce) J.J. Engel & R.M. Schust.	*Heteroscyphuspolychaetus* (Spruce) Hentschel & Heinrichs	Ecuador, Gradstein & Mandl, 10139 (GOET)	AM491296	–
*Chiloscyphusprofundus* (Nees) J.J.Engel & R.M.Schust.	*Lophocoleaprofunda* Nees	Germany, Hentschel Bryo, 01414 (JE)	AM282832	–
*Chiloscyphusprofundus* (Nees) J.J.Engel & R.M.Schust.	*Lophocoleaprofunda* Nees	Finland, 2000 & Piippo, 1470	–	AY149874
*Chiloscyphusrandii* (S.W.Arnell) J.J.Engel & R.M.Schust.	*Lophocolearandii* S.W. Arnell	Prince Edward Isles, Gremmen, 98-63 (JE)	AM282833	–
*Chiloscyphussabuletorum* (Hook.f. & Taylor) J.J.Engel & R.M.Schust.	*Lophocoleasabuletorum* (Hook. f. & Taylor) Gottsche, Lindenb. & Nees	Argentina, Hyvönen, 3233 (JE)	AM282834	–
*Chiloscyphussabuletorum* (Hook.f. & Taylor) J.J.Engel & R.M.Schust.	*Lophocoleasabuletorum* (Hook. f. & Taylor) Gottsche, Lindenb. & Nees	Chile, Busch et al. Bryo, 01396 (JE)	AM282835	–
*Chiloscyphussemiteres* (Lehm.) Lehm. & Lindenb.	*Lophocoleasemiteres* (Lehm.) Mitt.	Australia, Streimann, 58464 (GOET)	AM282836	–
*Chiloscyphussemiteres* (Lehm.) Lehm. & Lindenb.	*Lophocoleasemiteres* (Lehm.) Mitt.	The Netherlands, Stieperaere, 8611 (JE)	AM282837	–
*Chiloscyphussemiteres* (Lehm.) Lehm. & Lindenb.	*Lophocoleasemiteres* (Lehm.) Mitt.	New Zealand, Engel & von Konrat, 27982	–	FJ173301
*Chiloscyphusspinifer* (Hook.f. & Taylor) J.J.Engel & R.M.Schust.	*Cryptolophocoleaspinifera* (Hook. f. & Taylor) L. Söderstr.	New Zealand, Schäfer-Verwimp & Verwimp, 13808 (JE)	AM282838	–
*Chiloscyphusspinifer* (Hook.f. & Taylor) J.J.Engel & R.M.Schust.	*Cryptolophocoleaspinifera* (Hook. f. & Taylor) L. Söderstr.	New Zealand, Engel & von Konrat, 28452	–	FJ173302
*Chiloscyphustrachyopus* (Hook. f. & Taylor) Hässel	*Clasmatocoleatrachyopa* (Hook. f. & Taylor) Grolle	Chile, Hyvoenen, 5933 (GOET)	AM491298	–
*Chiloscyphusvermicularis* (Lehm.) Hässel	*Clasmatocoleavermicularis* (Lehm.) Grolle	Ecuador, Sauer & Gradstein, MS-E065 (GOET)	AM491299	–
*Clasmatocoleactenophylla* (Schiffn.) Grolle	*Clasmatocoleactenophylla* (Schiffn.) Grolle	Chile, Engel, 25779	–	FJ173304
*Clasmatocoleahumilis* (Hook.f. & Taylor) Grolle	*Clasmatocoleahumilis* (Hook.f. & Taylor) Grolle	Chile, Engel, 25274	–	FJ173305
*Clasmatocoleaobvoluta* (Hook.f. & Taylor) Grolle	*Clasmatocoleaobvoluta* (Hook.f. & Taylor) Grolle	Chile, Engel, 25696	–	FJ173306
*Cyanolophocoleaechinella* (Lindenb. & Gottsche) R.M. Schust.	*Heteroscyphusechinellus* (Lindenb. & Gottsche) J.J. Engel & X.L. He	New Zealand, Lewington, 1140 (H)	–	FJ919297
*Cyanolophocoleaechinella* (Lindenb. & Gottsche) R.M. Schust.	*Heteroscyphusechinellus* (Lindenb. & Gottsche) J.J. Engel & X.L. He	New Zealand, Engel, 27818 (F)	–	FJ919304
*Herbertusdicranus* (Taylor ex Gottsche, Lindenb. & Nees) Trevis.	*Herbertusdicranus* (Taylor ex Gottsche, Lindenb. & Nees) Trevis.	H3230549 (H)	KU523784	KU523718
*Heteroscyphusargutus* (Reinw., Blume & Nees) Schiffn.	*Heteroscyphusargutus* (Reinw., Blume & Nees) Schiffn.	Nepal, D.G. Long, 30333 (JE)	–	AY149861
*Heteroscyphusaselliformis* (Reinw., Blume & Nees) Schiffn.	*Heteroscyphusaselliformis* (Reinw., Blume & Nees) Schiffn.	Indonesia, Gradstein, 10240 (GOET)	AM180588	–
*Heteroscyphusbiciliatus* (Hook. f. & Taylor) J.J. Engel	*Heteroscyphusbiciliatus* (Hook. f. & Taylor) J.J. Engel	New Zealand, Frahm, 20-6 (GOET)	AM491300	–
*Heteroscyphuscoalitus* J.J. Engel	*Heteroscyphuscoalitus* J.J. Engel	Nepal, D.G. Long, 17402 (JE)	AM282839	–
*Heteroscyphuscoalitus* J.J. Engel	*Heteroscyphuscoalitus* J.J. Engel	Nepal, D.G. Long, 30316 (JE)	–	AY149865
*Heteroscyphuscuneistipulus* (Steph.) Schiﬀn.	*Heteroscyphuscuneistipulus* (Steph.) Schiﬀn.	New Zealand, Frahm, 9-15 (GOET)	AM282840	–
*Heteroscyphusfissistipus* (Hook.f. & Taylor) Schiffn.	*Heteroscyphusfissistipus* (Hook.f. & Taylor) Schiffn.	Ireland, D.G. Long, H4064 (JE)	AM282841	–
*Heteroscyphusinflatus* (Steph.) S.C. Srivast. & A. Srivast.	*Heteroscyphusinflatus* (Steph.) S.C. Srivast. & A. Srivast.	Nepal, D.G. Long, 30457 (JE)	–	AY149875
*Heteroscyphusplanus* (Mitt.) Schiffn.	*Heteroscyphusplanus* (Mitt.) Schiffn.	Japan, 1992 Mizutani, 15828	–	AY149872
*Heteroscyphussplendens* (Lehm. & Lindenb.) Grolle	*Heteroscyphussplendens* (Lehm. & Lindenb.) Grolle	Papua New Guinea, 1989 Hoffmann, 89-749	–	AY149876
*Heteroscyphuszollingeri* (Gottsche) Schiffn.	*Heteroscyphuszollingeri* (Gottsche) Schiffn.	China, 1998 Koponen et al. 57927	–	AY149879
*Hygrolembidiumacrocladum* (Berggr.) R.M. Schust.	*Hygrolembidiumacrocladum* (Berggr.) R.M. Schust.	Australia, Curnow, 5587	–	AY463560
*Leiomitralanata* (Hook.) R.M. Schust.	*Leiomitralanata* (Hook.) R.M. Schust.	New Zealand, Glenny s.n., 2001	–	AY463565
*Lepicoleaattenuata* (Mitt.) Steph.	*Lepicoleaattenuata* (Mitt.) Steph.	New Zealand, South Island, Stotler & Crandall-Stotler, 4586 (ABSH)	–	JF316578, AY507540
*Lepicoleaochroleuca* (Spreng.) Spruce	*Lepicoleaochroleuca* (Spreng.) Spruce	Chile, Hyvonen, 2938	–	AY463566
*Lepicoleascolopendra* (Hook.) Dumort. ex Trevis.	*Lepicoleascolopendra* (Hook.) Dumort. ex Trevis.	Australia, Streimann, 55445	–	AY463568
*Leptophyllopsislaxa* (Mitt.) Hamlin	*Leptophyllopsislaxa* (Mitt.) Hamlin	Australia, Streimann, 43810 (JE)	AM491301	–
*Leptoscyphusamphibolius* (Nees) Grolle	*Leptoscyphusamphibolius* (Nees) Grolle	Brazil, Schafer-Verwimp, Schafer 14748	–	EU350474
*Leptoscyphusgibbosus* (Taylor) Mitt.	*Leptoscyphusgibbosus* (Taylor) Mitt.	Dominican Republic, Schafer-Verwimp (herb. Schafer), 17647	–	DQ176702
*Leptoscyphusgibbosus* (Taylor) Mitt.	*Leptoscyphusgibbosus* (Taylor) Mitt.	Costa Rica, Herbarium Schafer-Verwimp, SV/H-0364	–	EU350480
*Leptoscyphusporphyrius* (Nees) Grolle	*Leptoscyphusporphyrius* (Nees) Grolle	Ecuador, Schafer-Verwimp (herb. Schafer), 23229/a	–	DQ176707
*Leptoscyphusporphyrius* (Nees) Grolle	*Leptoscyphusporphyrius* (Nees) Grolle	Ecuador, Herbarium Schafer-Verwimp, Schafer 24214/a	–	EU350481
*Lophocoleabidentata* (L.) Dumort.	*Lophocoleabidentata* (L.) Dumort.	Poland: Silesian upland, K. Jedrzejko & A. Stenel (W-58)	–	AY149862
*Lophocoleacuspidata* (Nees) Limpr.	*Lophocoleabidentata* (L.) Dumort.	China, Hunan Province, Sang-Zhi Co., Koponen et al. 48430	–	AY149866
*Lophocoleaheterophylla* (Schrad.) Dumort.	*Lophocoleaheterophylla* (Schrad.) Dumort.	USA, Indiana, ML Sargent’s culture collection, #481	–	AF231899
*Lophocoleamartiana* Nees	*Cryptolophocoleamartiana* (Nees) L. Söderstr., Crand.-Stotl. & Stotler	French Guiana, Kourou, Gradstein, 6265	–	AY149870
*Lophocoleasikkimensis (Steph.) Herzog & Grolle	*Cryptolophocoleasikkimensis (Steph.) Bakalin et Maltseva	Vietnam, Lao Cai Province, V.A. Bakalin & K.G. Klimova, V-12-17-17 (VBGI)	**OK523503**	**OK562106**
**Lophocoleasikkimensis (Steph.) Herzog & Grolle	**Cryptolophocoleasikkimensis (Steph.) Bakalin et Maltseva	Vietnam, Lao Cai Province, V.A. Bakalin, V-3-86-16 (VBGI)	**OK523504**	–
*Mastigophorawoodsii* (Hook.) Nees	*Mastigophorawoodsii* (Hook.) Nees	China, D.Long, 33696 (E)	–	JF316581
*Mastigophorawoodsii* (Hook.) Nees	*Mastigophorawoodsii* (Hook.) Nees	Australia, Frahm, CANB639918, 2000	–	AY463574
*Pachyglossatenacifolia* (Hook. f. & Taylor) Herzog & Grolle	*Pachyglossatenacifolia* (Hook. f. & Taylor) Herzog & Grolle	New Zealand, Bartlett 196 (JE)	AM491297	–
*Pedinophylluminterruptum* (Nees) Kaal.	*Pedinophylluminterruptum* (Nees) Kaal.	Germany, Schaefer-Verwimp, 35485 (M)	KT992498	–
*Pedinophylluminterruptum* (Nees) Kaal.	*Pedinophylluminterruptum* (Nees) Kaal.	Russia, N.A. Konstantinova & A.N. Savchenko, k508/7-07 (F)	–	KJ802073
*Plagiochilaalternans* Lindenb. & Gottsche	*Plagiochilaalternans* Lindenb. & Gottsche	Bolivia, Heinrichs et al. GP 16 (GOET)	AY550130	–
*Plagiochilaasplenioides* (L.) Dumort.	*Plagiochilaasplenioides* (L.) Dumort.	Finland, Nuuksio National Park, He-Nygren and Piippo 1467	–	AY149858
*Plagiochilafruticella* (Hook. f. & Taylor) Gottsche, Lindenb. & Nees	*Plagiochilafruticella* (Hook. f. & Taylor) Gottsche, Lindenb. & Nees	New Zealand, Engel & von Konrat, 23943 (GOET)	AM180613	–
*Plagiochilapleurata* (Hook. f. & Taylor) Gottsche, Lindenb. & Nees	*Plagiochilapleurata* (Hook. f. & Taylor) Gottsche, Lindenb. & Nees	New Zealand, Schaefer-Verwimp & Verwimp, 13777 (GOET)	AM180615	–
*Plagiochilaporelloides* (Torr. ex Nees) Lindenb.	*Plagiochilaporelloides* (Torr. ex Nees) Lindenb.	Germany, Schaefer-Verwimp, 31077 (M)	KX896587	–
*Plagiochilaporelloides* (Torr. ex Nees) Lindenb.	*Plagiochilaporelloides* (Torr. ex Nees) Lindenb.	USA, Alaska, B. Shaw, F955/1 (DUKE)	–	KF943056
*Plagiochilasichotensis* Bakalin & Vilnet	*Plagiochilasichotensis* Bakalin & Vilnet	Russia, Russian Far East, Primorsky Territory, V.A. Bakalin & G.A. Arutinov, Arutinov 1-25-13 (VBGI)	MF947695	MF947697
*Plagiochilaxerophila* Bakalin & Vilnet	*Plagiochilaxerophila* Bakalin & Vilnet	China, Sichuan Province, V.A. Bakalin & K.G. Klimova, China-46-2-17 (VBGI)	–	MK123266
*Tetracymbaliellacymbalifera* (Hook. f. & Taylor) Grolle	*Tetracymbaliellacymbalifera* (Hook. f. & Taylor) Grolle	New Zealand, M.A.M. Renner, 6139 (NSW)	KT992470	–
*Tetracymbaliellacymbalifera* (Hook. f. & Taylor) Grolle	*Tetracymbaliellacymbalifera* (Hook. f. & Taylor) Grolle	New Zealand, Frahm 1-23 (MO-5131915)	–	DQ026625
*Triandrophyllumsubtrifidum* (Hook. f. & Taylor) Fulford & Hatcher	*Triandrophyllumsubtrifidum* (Hook. f. & Taylor) Fulford & Hatcher	Bolivia, Churchill et al. 22800	AJ972455	–
*Triandrophyllumsubtrifidum* (Hook. f. & Taylor) Fulford & Hatcher	*Triandrophyllumsubtrifidum* (Hook. f. & Taylor) Fulford & Hatcher	Venezuela, Ricardi, 9730/T	–	JF316580
*Trichocoleatomentella* (Ehrh.) Dumort.	*Trichocoleatomentella* (Ehrh.) Dumort.	China, He-Nygren, 1137	–	AY463590
*Trichotemnomacorrugatum* (Steph.) R.M. Schust.	*Trichotemnomacorrugatum* (Steph.) R.M. Schust.	New Zealand, Glenny 8426	–	AY463591
*Zoopsisargentea* (Hook. f. & Taylor) Gottsche, Lindenb. & Nees	*Zoopsisargentea* (Hook. f. & Taylor) Gottsche, Lindenb. & Nees	Australia, Streimann, 51704	–	AY463595
*Zoopsisargentea* (Hook. f. & Taylor) Gottsche, Lindenb. & Nees	*Zoopsisargentea* (Hook. f. & Taylor) Gottsche, Lindenb. & Nees	New Zealand, J.J.Engel, 23962	–	JF316577

DNA isolation, amplification, and sequencing. DNA was extracted from dried liverwort tissues using the NucleoSpin Plant II Kit (Macherey-Nagel, Germany). Ampliﬁcation of ITS1–2, *trn*L-F, and the *trn*G-intron was performed using an Encyclo Plus PCR kit (Evrogen, Moscow, Russia) with the primers listed in Table 2.

### ﻿DNA isolation, amplification, and sequencing

DNA was extracted from dried liverwort tissues using the NucleoSpin Plant II Kit (Macherey-Nagel, Germany). Ampliﬁcation of ITS1–2, *trn*L-F, and the *trn*G-intron was performed using an Encyclo Plus PCR kit (Evrogen, Moscow, Russia) with the primers listed in Table 2.

**Table 2.** Primers used in polymerase chain reaction (PCR) and cycle sequencing.

Locus	Sequence (5’-3’)	Direction	Annealing temperature (°C)	Reference
ITS 1–2 nrDNA	CGTTGTGAGAAGTTCATTAAACC	forward	64	Feldberg et al. 2016
ITS 1–2 nrDNA	GATATGCTTAAACTCAGCGG	reverse	58	Milyutina et al. 2010
*trn*L-F cpDNA	CGAAATTGGTAGACGCTGCG	forward	62	Bakalin et al. 2021
*trn*L-F cpDNA	ATTTGAACTGGTGACACGAG	reverse	58	Taberlet et al. 1991
*trn*G-intron cpDNA	ACCCGCATCGTTAGCTTG	forward	56	Pacak and Szweykowska-Kulinska 2000
*trn*G-intron cpDNA	GCGGGTATAGTTTAGTGG	reverse	54	Pacak and Szweykowska-Kulinska 2000

The polymerase chain reaction was performed in a total volume of 20 µl, including 1 µl of template DNA, 0.4 µl of Encyclo polymerase, 5 µl of Encyclo buffer, 0.4 µl of dNTP-mixture (included in Encyclo Plus PCR Kit), 13.4 µl (for *trn*L-F and the *trn*G-intron)/12.4 µl (for ITS1–2) of double-distilled water (Evrogen, Moscow, Russia), 1 µl of dimethylsulfoxide/DMSO (for ITS1–2) and 0.4 µl of each primer (forward and reverse, at a concentration of 5 pmol/µl). Polymerase chain reactions were carried out using the following program: 180 s initial denaturation at 95 °C, followed by 30–40 cycles of 30 s denaturation at 94 °C, 20 (for *trn*L-F) – 30 s (for ITS1–2, *trn*G-intron) annealing at 56 °C (*trn*G-intron) or 58 °C (*trn*L-F and ITS1–2), and 30 s elongation at 72 °C. Final elongation was carried out in one 5-min step at 72 °C. Amplified fragments were visualized on 1% agarose TAE gels by EthBr staining and purified using the Cleanup Mini Kit (Evrogen, Moscow, Russia). The DNA was sequenced using the BigDye Terminator v. 3.1 Cycle Sequencing Kit (Applied Biosystems, USA) with further analysis of the reaction products following the standard protocol on an ABI Prism 3100-Avant Genetic Analyser (Applied Biosystems, USA) in the Genome Center (Engelhardt Institute of Molecular Biology, Russian Academy of Sciences, Moscow).

### ﻿Phylogenetic analyses

The datasets were produced for the ITS1–2 and *trn*L-F loci. Both datasets were aligned using MAFFT (Katoh and Standley 2013) with standard settings and then edited manually in BioEdit ver. 7.2.5 (Hall 1999). All positions of the final alignments were included in the phylogenetic analyses.

Phylogenies were reconstructed under three criteria: maximum parsimony (MP) with Mega X (Kumar et al. 2018), maximum likelihood (ML) with IQ-tree ver. 1.6.12 (Nguyen et al. 2015) and Bayesian inference (BA) with MrBayes ver. 3.2.7 (Ronquist et al. 2012).

MP analysis for both datasets included 1,000 bootstrap replicates, default settings for all other parameters, and treated gaps as partial deletions with a site coverage cut-off of 95%.

For the ML analysis, the best fitting evolutionary model of nucleotide substitutions according to the BIC value was TIM3+F+I+G4 for the ITS dataset and TVM+F+I+G4 for the *trn*L-F dataset as determined by IQ-tree. Consensus trees were constructed with 1000 bootstrap replicates.

Indels for both datasets were coded with FastGap ver. 1.2 (Borchsenius 2009) and then added to the nucleotide matrices in the Bayesian analyses. Bayesian analyses were performed by running two parallel analyses using the GTR+I+G model. For both datasets, the analysis consisted of four Markov chains. Chains were run for five million generations, and trees were sampled every 500^th^ generation. The first 2,500 trees in each run were discarded as burn-in; thereafter, 15,000 trees were sampled from both runs. Bayesian posterior probabilities were calculated from the trees sampled after burn-in. The average standard deviation of split frequencies between two runs was 0.0017 for ITS1–2 and 0.0068 for *trn*L-F.

The infrageneric and infraspecific variability of ITS1–2 and *trn*L-F were quantified as the average pairwise *p*-distances calculated in Mega X (Kumar et al. 2018) using the pairwise deletion option for counting gaps.

## ﻿Results

Five new sequences from *Lophocoleasikkimensis* specimens were deposited in GenBank: two for ITS1–2, one for *trn*L-F and two for *trn*G-intron cpDNA. ITS1–2 alignment of the 55 specimens consisted of 955 character sites, and the *trn*L-F alignment of 53 specimens consisted of 612 character sites. The parameters of the tested alignments are shown in Table 3.

**Table 3.** The characteristics of ITS1–2, and *trn*L-F nucleotide sequence alignments.

Locus	Total sites	Conservative sites		Variable sites		Parsimony-informative sites	
		base pairs	%	base pairs	%	base pairs	%
ITS1–2	955	376	39.37	376	39.37	431	45.13
*trn*L-F	612	325	53.11	325	53.11	208	33.99

The MP analysis for ITS1–2 yielded a single parsimonious tree with CI = 0.364388 and RI = 0.619946. The ML criterion recovered a bootstrap consensus tree with a log-likelihood = -10978.46. The arithmetic means of the log likelihoods in Bayesian analysis for each sampling run were -11026.6 and -11028.06.

The MP analysis for *trn*L-F yielded five equally parsimonious trees with CI = 0.480315 and RI = 0.697248. The ML criterion recovered a bootstrap consensus tree with a log-likelihood = -4951.09. The arithmetic means of log likelihoods in the Bayesian analysis for each sampling run were -4993.95 and -4989.08.

**Figure 1.**
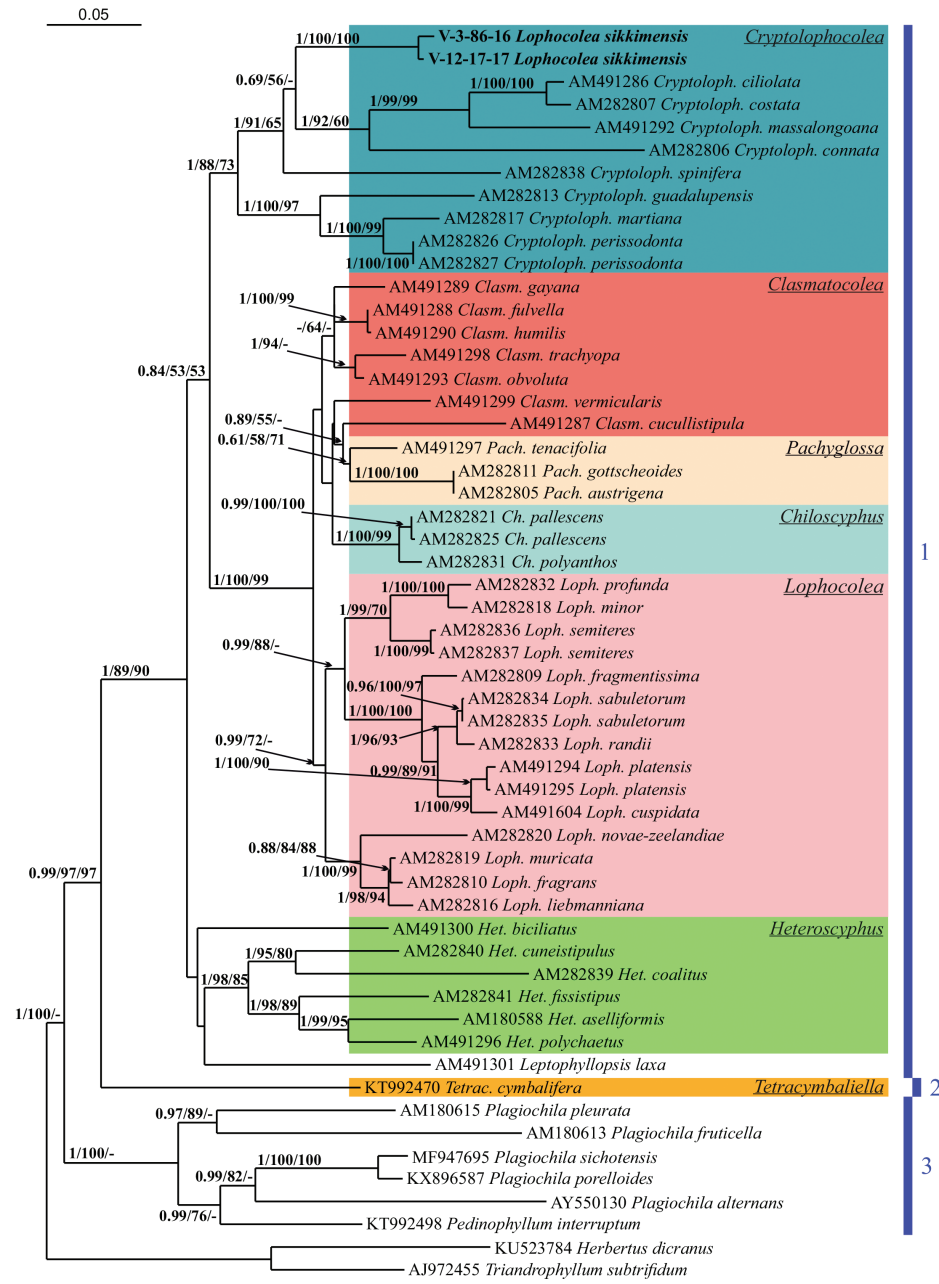
Phylogram obtained in a Bayesian analysis for the genus *Cryptolophocolea* and related taxa based on ITS1–2 dataset. The values of Bayesian posterior probabilities and bootstrap support from the MP and ML analyses greater than 0.50 (50%) are indicated. Taxon names and GenBank accession numbers are provided. Newly studied specimens are marked in bold **1** family Lophocoleaceae**2** family Brevianthaceae**3** family Plagiochilaceae.

**Figure 2.**
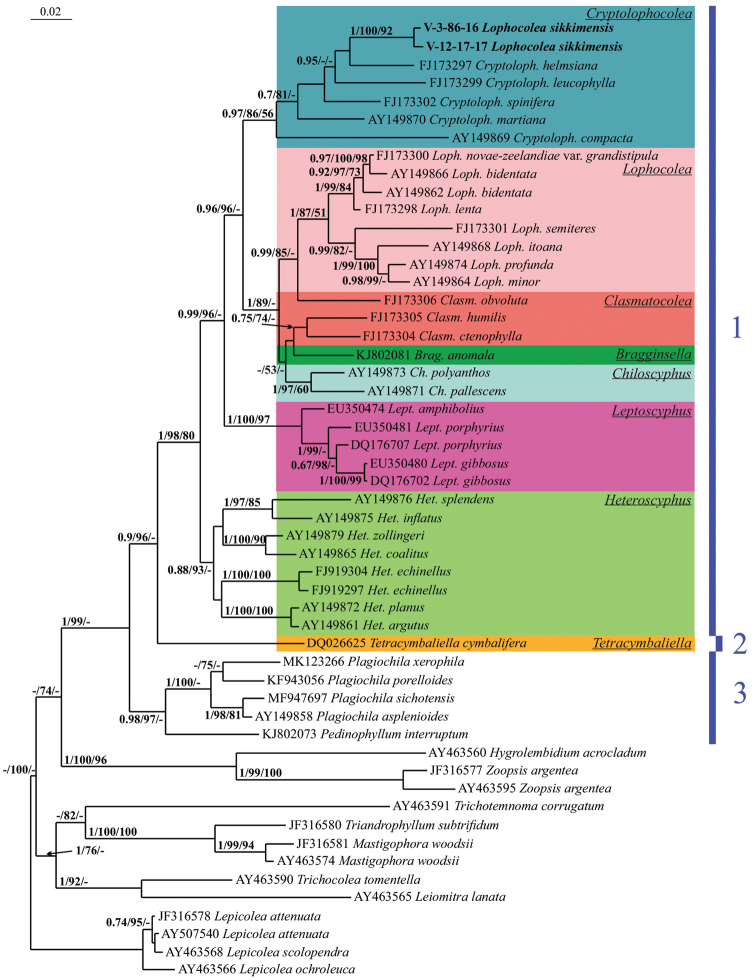
Phylogram obtained in a Bayesian analysis for the genus *Cryptolophocolea* and related taxa based on *trn*L-F dataset. The values of Bayesian posterior probabilities and bootstrap support from the MP and ML analyses greater than 0.50 (50%) are indicated. Specimen names and GenBank accession numbers are provided. Newly studied specimens are marked in bold **1** family Lophocoleaceae**2** family Brevianthaceae**3** family Plagiochilaceae.

The trees constructed for each dataset by the three different methods appeared highly congruent. Fig. 1 shows the phylogenetic tree based on the ITS1–2 dataset retained under Bayesian analysis, along with bootstrap support (BS) values from the MP and ML analyses and the Bayesian posterior probabilities (PP) for each node. Fig. 2 shows the BA tree based on the *trn*L-F dataset as well as the BS from the MP and ML calculations and the PP for each node.

The topologies obtained here are quite similar to previously published phylogenies in the reinstatement of Lophocoleaceae (Hentschel et al. 2006a), the identification of the systematic position of *Pachyglossa* and *Clasmatocolea* by Hentschel et al. (2007), and the reconstruction of the phylogeny of Lophocoleaceae-Plagiochilaceae-Brevianthaceae by Patzak et al. (2016). The two studied specimens of *Lophocoleasikkimensis* formed a strongly supported subclade in both calculations (100/100/1.00, 1/100/92 Figs 1, 2), which was placed within robustly supported clade of the genus *Cryptolophocolea* (1/88/73 in ITS1–2, 0.97/86/56 in *trn*L-F). The position of *Clasmatocoleaobvoluta* was unstable: based on the *trn*L-F reconstruction, it belongs in *Lophocolea*, but based on the ITS1–2 sequences, it should remain in *Clasmatocolea*, possibly, it could be associated with sequence origin from different specimens and their identification.

The intergroup average distance between *Lophocoleasikkimensis* and *Cryptolophocolea* (Table 4) is lower than most of its distances to other genera of Lophocoleaceae (although the average infrageneric distance in *Cryptolophocolea* is maximum compared to other Lophocoleaceae genera).

**Table 4.** Inter- and Infrageneric *p*-distances, ITS1–2 and *trn*L-F. The number of base differences per site from averaging over all sequence pairs within and between each group are shown. The upper triangle – data for ITS 1–2, the lower triangle – data for *trn*L-F; — – data are absent.

*trn*L-F, %	Taxon	*Lophocoleasikkimensis*+ *Cryptolophocolea*	*Lophocoleasikkimensis*	*Cryptolophocolea*	*Lophocolea*	*Clasmatocolea*	*Chiloscyphus*	*Leptoscyphus*	*Heteroscyphus*	*Pachyglossa*	ITS 1–2, %
0.061	*Lophocoleasikkimensis*+*Cryptolophocolea*		–	–	0.164	0.150	0.153	–	0.167	0.156	–
0	*Lophocoleasikkimensis*	–		**0.128**	0.142	0.129	0.130	–	0.154	0.138	0.011
**0.070**	*Cryptolophocolea*	–	**0.058**		0.168	0.155	0.158	–	0.170	0.161	**0.135**
0.040	*Lophocolea*	0.092	0.085	0.094		0.088	0.096	–	0.163	0.100	0.074
0.058	*Clasmatocolea*	0.082	0.069	0.088	0.068		0.074	–	0.146	0.075	0.063
0.037	*Chiloscyphus*	0.081	0.069	0.087	0.062	0.056		–	0.147	0.079	0.013
0.021	*Leptoscyphus*	0.086	0.081	0.087	0.094	0.086	0.090		–	–	–
0.055	*Heteroscyphus*	0.088	0.080	0.091	0.091	0.085	0.083	0.074		0.151	0.135
–	*Pachyglossa*	–	–	–	–	–	–	–	–		0.044

Therefore, according to the estimated phylogenetic relationships and level of genetic differences, *Lophocoleasikkimensis* should be transferred to the genus *Cryptolophocolea*.

Due to the obvious position of the studied specimens in the *Cryptolophocolea* clade, we provide the corresponding new combination for *Lophocoleasikkimensis*:

### ﻿ Cryptolophocolea sikkimensis

Taxon classificationPlantaeJungermannialesLophocoleaceae

(Steph.) Bakalin & Maltseva comb. nov.

06315E81-EC50-5B2D-8B98-02D8FF23129C

#### Basionym.

*Herpocladiumsikkimense* Steph., Sp. Hepat. (Stephani) 6: 349, 1922 (=*Lophocoleasikkimensis* (Steph.) Herzog & Grolle, Rev. Bryol. Lichénol. 27 (3/4): 164, 1958 [1959])

## ﻿Discussion

### ﻿Morphology

Söderström et al. (2013) list 20 genera in Lophocoleaceae, excluding *Conoscyphus*, which was later transferred to Acrobolbaceae by Dimon et al. (2018). The genera in this large and morphologically variable family have several common features (with several exceptions), including generally obliquely to very obliquely inserted leaves, rhizoids mostly from the small-celled area near the underleaf bases (thus the stem is free of rhizoids), trigonous perianth (exceptions are common) and generally bilobed leaves (the entire leaves are sparsely distributed across the family). *Cryptolophocoleasikkimensis* is distinguished by generally entire underleaves that are widely connate on both sides with the leaves. These leaves (along with entire leaves), generally occur in *Heteroscyphus*, the basal clade of *Lophocoleaceae* (Figs 1, 2). However, the entire leaves of *Cryptolophocoleasikkimensis* are quite different from the entire leaves in *Heteroscyphus*. In *Heteroscyphus*, *Chiloscyphus*, *Cryptolophocolea* and *Lophocolea*, which generally have entire leaves, the leaves are lingulate but not ovate, with an apiculate to obtuse apex. The leaf shape of *Cryptolophocoleasikkimensis* is therefore similar to the leaf shape of *Cuspidatulacontracta* (Reinw., Blume & Nees) Steph. (Adelanthaceae) and is unlikely to occur in Lophocoleaceae.

**Figure 3.**
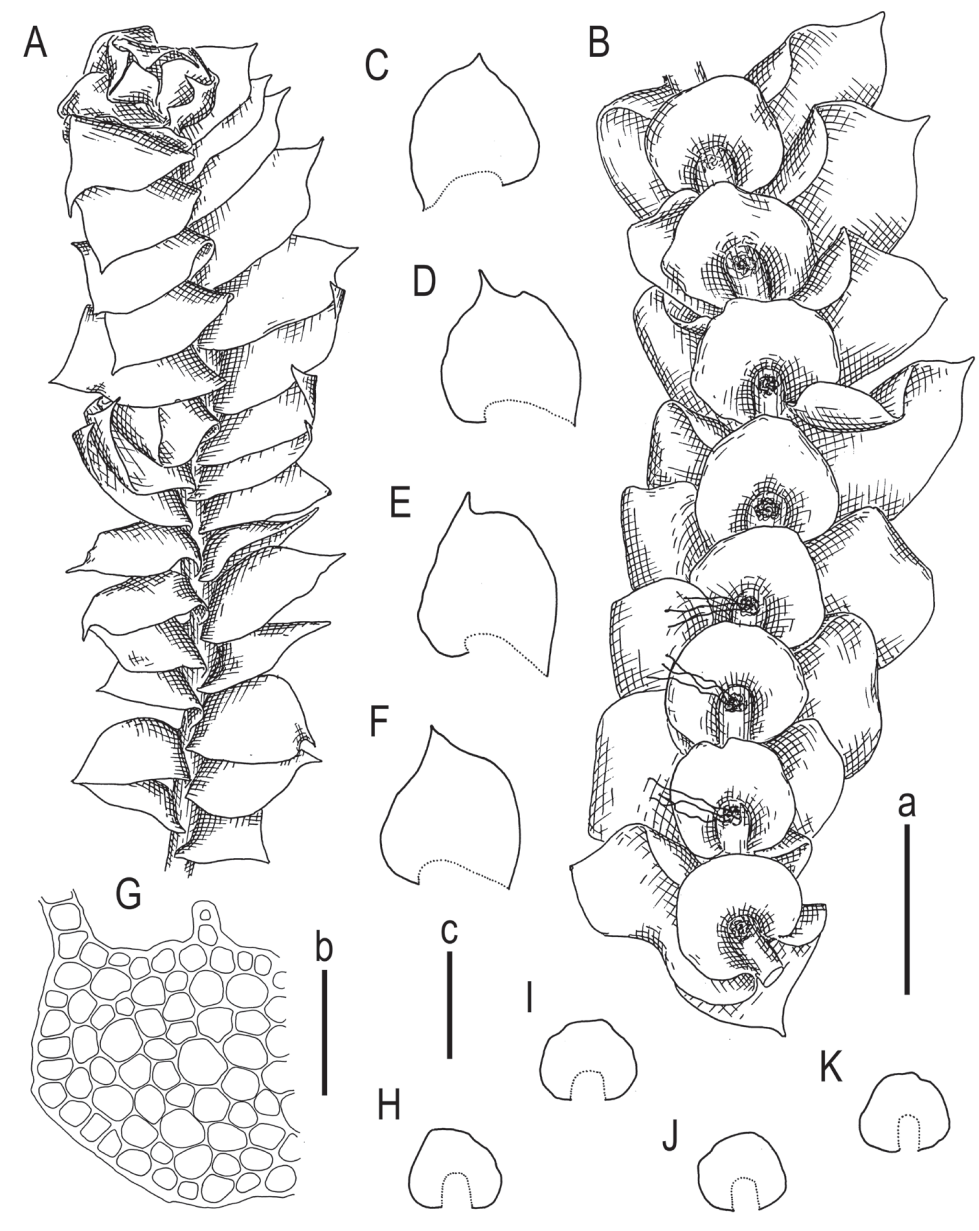
*Cryptolophocoleasikkimensis* (Steph.) Bakalin & Maltseva, comb. nov. **A** plant habit, fragment, dorsal view **B** plant habit, fragment, ventral view **C–F** leaves **H–K** underleaves **G** stem cross-section, fragment. Scale bars: 1 mm **a** (**A, B**); 100 µm **b** (**G**); 1 mm **c** (**C–F, H–K**). All from V-8-54-17 (VBGI).

However, the morphology of *Cryptolophocoleasikkimensis* is highly variable, and along with well-developed plants with ovate leaves and entire underleaves, modifications with shortly bilobed to bidentate leaves and underleaves may be observed. Indeed, Kitagawa (1974: 36) wrote, “Plants of Taiwan (Fig. 3) are so markedly different from the typical ones that I felt some hesitation in regarding them as conspecific with the Himalayan plants. Most leaves are distinctly bilobed, and underleaves are often emarginate”. These features may have an atavistic nature.

Notably, the Vietnamese populations, located between the extreme flanks of the species range, sometimes exhibit an intermediate morphology: plants with bilobed leaves and emarginate underleaves are often found. However, these plants are usually smaller than the well-developed individuals and characterized by distanced leaves and underleaves; they generally provide an impression of weakly developed or “suppressed” shoots. This intermediate morphology corresponds to the observations by Kitagawa (1974) that plants from Thailand are characterized by a smaller size. Our well-developed plants are distinctly larger, and the mats from drier habitats contain smaller plants. Additionally, the rounded apex of the leaf is frequently found in Himalayan plants. In contrast, Indochinese plants (both from Thailand and Vietnam) as well as Bornean plants (according to Kitagawa (1974) and our own observations) never exhibit a rounded apex; instead, the leaf apex is generally acute (rarely bicuspidate in smaller plants). Thus, the observed morphological variability, clearly correlating with geographic longitude, is likely associated with genetic infraspecific variability. However, we can neither prove nor disprove this assumption.

### ﻿Ecology

Kitagawa (1974: 35) noted that “Plants occur on various substrata (rocks, rotten logs, humus) but usually do not grow directly on such substrata but creeping larger bryophytes”. In general, the same can be said about the ecological preferences of Vietnamese plants. Meanwhile, it should be noted that in Thailand (in the apical part of Mt. Doi Inthanon in Chiangmai Province, where the species is observed), known specimens of the species (only two, both cited by Kitagawa 1974) are restricted to tree branches and trunks (Kitagawa 1974). Moreover, the species is confined to stony substrates in its only locality in Yunnan, adjacent to Indochina from the north, where it was found in the vicinity of Lijiang (Piippo et al. 1998). Thus, the known variation in habitat in Vietnam exceeds that known in China’s Yunnan Province and Thailand and corresponds to the general variation across species range (detailed information is included in the specimens examined section). The species associates in Vietnam include a lot of liverwort taxa, such as *Mniolomafuscum* (Lehm.) R.M. Schust., *Scapaniaciliatospinosa* Horik., Lepidoziacf.subtransversa Steph., *Herbertusdicranus* (Gottsche, Lindenb. & Nees) Trevis. and *Riccardia* sp.

### ﻿Description (based on plants from Vietnam)

**Figure 4.**
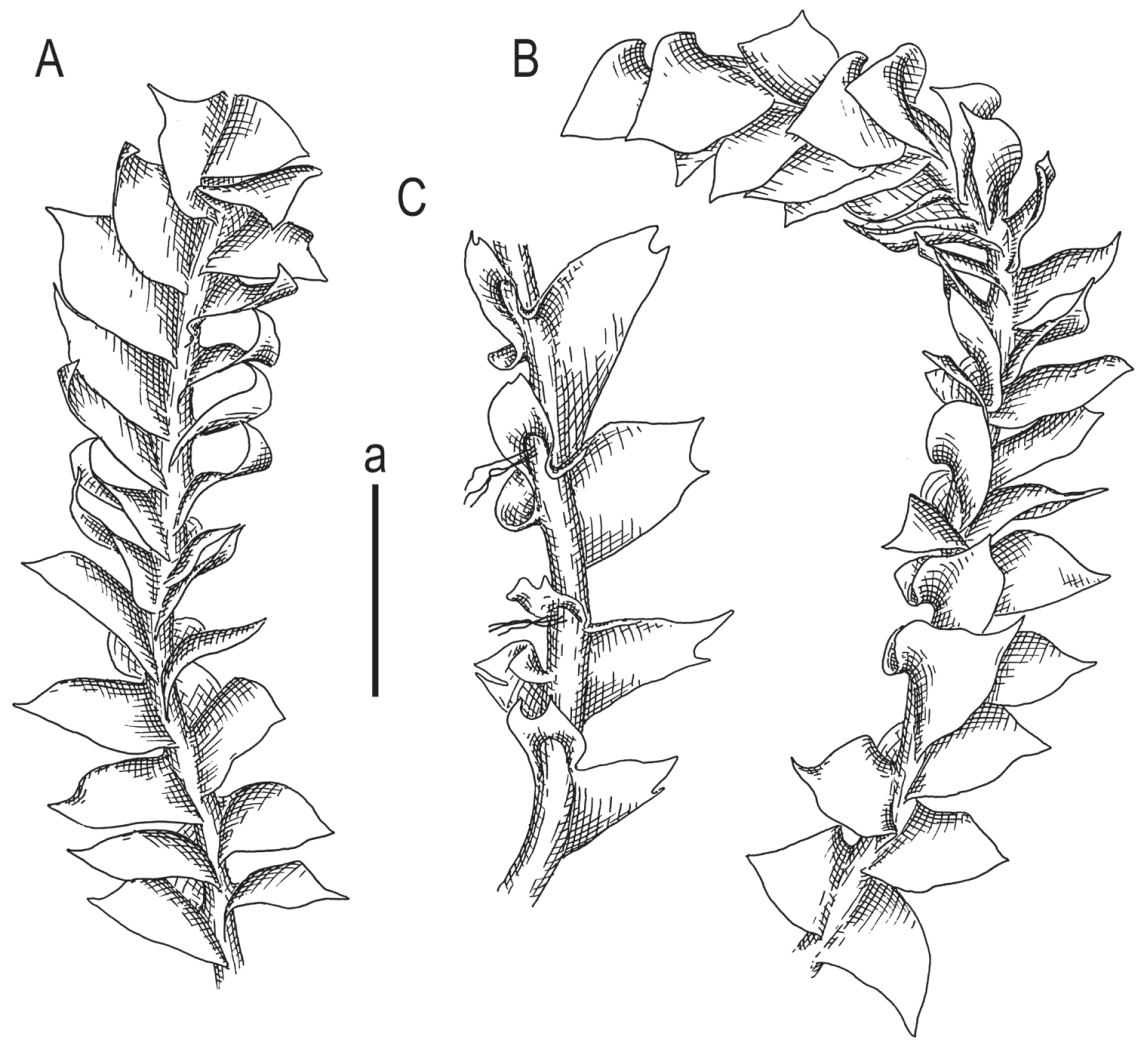
*Cryptolophocoleasikkimensis* (Steph.) Bakalin & Maltseva, comb. nov. **A, B** plant habit, fragment, dorsal view **C** plant habit, fragment, ventral view. Scale bars: 1 mm **a** (**A–C**). All from V-8-54-17 (VBGI).

**Figure 5.**
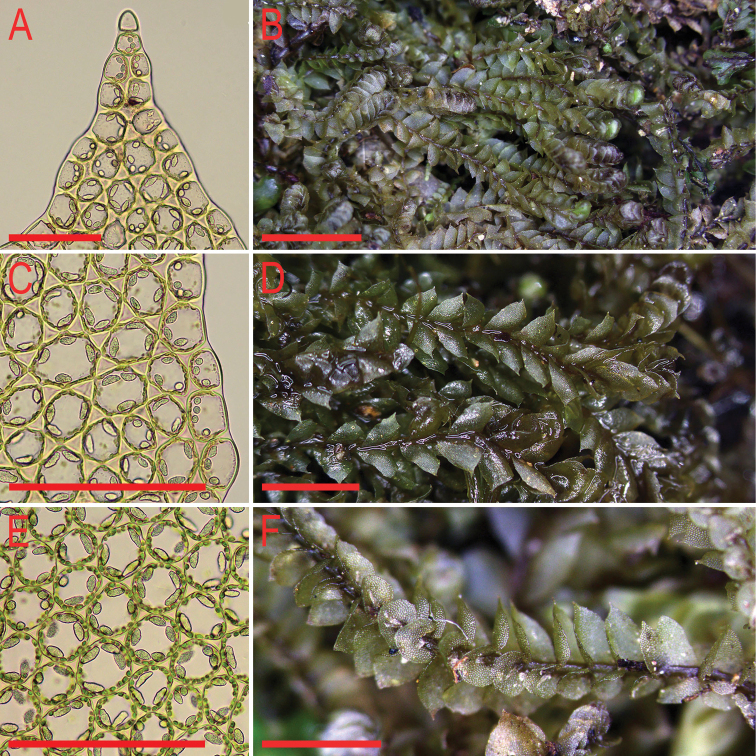
*Cryptolophocoleasikkimensis* (Steph.) Bakalin & Maltseva, comb. nov. **A** oil bodies in apical part of the leaf **B** mat **C** oil bodies in leaf margin cells **D** shoots, fragment, dorsal view **E** oil bodies in midleaf cells **F** shoot, fragment, ventral view. Scale bars: 100 µm (**A, C, E**); 5 mm (**B**); 2 mm (**D, F**). All from V-8-54-17 (VBGI).

Plants yellowish green, greenish and whitish yellowish to yellowish brownish, sometimes grading to grayish brownish in the herbarium, gentle, very fragile and glistening when dry, forming loose pure patches over other bryophytes or rarely intermixed with *Riccardia* sp., *Herbertusdicranus*, *Plicanthus*, *Scapaniaciliatospinosa*, *Mniolomafuscum*; creeping to loosely ascending (very rarely suberect in dense patches); normally developed shoots 1.1–2.0 mm wide (narrower, depauperate plants are commonly occurring) and 8–20(–30) µm long. Rhizoids regular, in erect or upraise spreading fascicles, originating from a small-celled initial zone of the underleaf adjacent to the stem in the axial part of the underleaf, fascicles 0.1–0.5 mm long. Stem rarely intercalary (lateral, from the middle part of the sinus) branched; cross section slightly transversely ellipsoidal, ca. 170–200 × 220–250 μm, external wall distinctly thickened, cell in 1(–2) marginal rows thick-walled, with large (sometimes loosely confluent) concave trigones, 17–27 μm in diameter, inner cells thin-walled, trigones moderate to large, concave, 23–27 μm in diameter. Leaves contiguous to distant in depauperate shoots, obliquely spreading, very obliquely to obliquely inserted (insertion line 20–45° with stem axis), barely decurrent dorsally, very ventral end of the insertion line subtransverse, dorsally leaves alternate to subopposite with a somewhat adjacent one to another dorsal bases, ventrally widely connate with underleaves; in general outline slightly convex to concave (never flat), with leaf apex commonly turned to the apical part of the shoot, when flattened in the slide widely ovate to obliquely ovate and widely ovate-triangular, widest very near to the base, apex acute to apiculate, rarely shortly bilobed with unequal to subequal lobes (bilobed apex mostly present in small shoots), normally developed leaves 900–1200 × 950–1100 μm. Underleaves loosely canaliculate, if looking from the ventral side, widely connate with leaves in both sides, transversely ellipsoidal, with apex entire, rarely emarginate to shortly bilobed (with sinus semicrescentic), insertion line arcuate (sinuate), 400–600 × 700–850 μm. Midleaf cells subisodiametric, 22–33 μm in diameter or shortly oblong, to 38 μm long, thin-walled, trigones large, mostly triangle to slightly concave or slightly convex, cuticle virtually smooth; cells along leaf margin subisodiametric (subquadrate), 21–25 μm in diameter to slightly elongate along the margin, to 25–27 μm long; oil bodies in the midleaf cells 2–5 per cell, finely granulate, irregularly oblong, ellipsoidal to shortly fusiform, 8–17 × 5–7(–8) μm, grayish (Figs 3–5).

**Specimens examined (North Vietnam).** Vietnam • Lao Cai Province, Sa Pa District, San Sa Ho Commune, Hoang Lien Range, Hoang Lien National Park, one of the ways to the Phan Xi Pan Peak; 22°18.8'N, 103°45.933'E; 2727 m a.s.l.; 3 Apr. 2018; V.A. Bakalin & K.G. Klimova leg.; thickets of *Sinobambusa* with many rocky outcrops and *Rhododendron* trees, partly shaded moist cliff, over *Sphagnum* mat; VBGI V-16-6-18 • same collection data as for preceding; 22°19.2'N, 103°46.183'E; 2610 m a.s.l.; 22 Apr. 2017; V.A. Bakalin & K.G. Klimova leg.; evergreen south subtropical mountain forest with bamboo thickets and many rocky outcrops, open moist cliff; VBGI V-12-17-17 • same collection data as for preceding; 22°18.45'N, 103°46.567'E; 2900 m a.s.l.; 20 Apr. 2017; V.A. Bakalin & K.G. Klimova leg.; *Rhododendron* dominated forest with bamboo thickets and many rocky outcrops, moist cliff in part shade; VBGI V-9-22-17; • same collection data as for preceding; 22°18.25'N, 103°46.5'E; 3050 m a.s.l.; 20 Apr. 2017; V.A. Bakalin & K.G. Klimova leg.; *Rhododendron* dominated forest with bamboo thickets and many rocky outcrops, moist open cliffs; VBGI V-8-14-17, V-8-29-17, V-8-32-17, V-8-52-17, V-8-53-17, V-8-54-17, V-8-62-17, V-8-73-17, V-8-13-17 • same collection data as for preceding; 22°18.183'N, 103°46.517'E; 3100 m a.s.l.; 17 Mar. 2016; V.A. Bakalin leg.; evergreen south subtropical mountain forest over the peak, partly shaded moist cliffs; VBGI V-3-86-16, V-3-91-16, V-3-61-16, V-3-81-16 • same collection data as for preceding; Lai Châu Province, Ta Leng Commune, Pu Ta Leng Mt. summit; 22°25.367'N, 103°36.233'E; 3050 m a.s.l.; 30 Mar. 2018; V.A. Bakalin & K.G. Klimova leg.; rhododendron trees with a dense bamboo understory, partly shaded moist decaying decorticated fallen tree trunk; VBGI V-11-45-18, V-11-16-18 • same collection data as for preceding; partly shaded mesic trunk of a living tree; VBGI V-11-36-18.

### ﻿Distribution

*Cryptolophocoleasikkimensis* has a pronounced Sino-Himalayan distribution. Its range stretches from Nepal to Taiwan and Borneo. Specifically, the species is found in China (Yunnan and Taiwan Provinces), North Borneo, Bhutan, Nepal, India (Sikkim, Darjeeling), North Thailand, and Vietnam (Kitagawa 1974; Long and Grolle 1990; Piippo 1990; Piippo et al. 1998; Bakalin et al. 2018; the present paper). Thus, the species ranges from the “*Rhododendron* flora” and “*Metasequoia* flora” (Wu and Wu 1996; Chen et al. 2018) in East Asia, to the Indochinese floristic region in the Palaeotropics (although in the upper mountain belts in areas with widely distributed Sino-Himalayan taxa) and to the Malesian floristic region. The reports from the Malesian region (both specimens cited by Kitagawa are from Kinabalu Mt.) are restricted to the upper belts (2900–3000 m a.s.l.), but distant from the area with widely distributed Sino-Himalayan taxa. The distribution of *Cryptolophocoleasikkimensis* in Taiwan is far less surprising. This island has, in a floristic sense, very close relationships with the Sino-Himalayan flora, and is the eastern outpost for some exclusively (or at least predominantly) Himalayan species: *Acrobolbusciliatus*, *Anastreptaorcadensis*, *Bazzaniaimbricata*, *Bazzaniasikkimensis*, *Frullaniagaudichaudii*, *Gymnomitrionrubidum*, *Odontoschismagrosseverrucosum*, and many others (Wang et al. 2011).

As described in the following section, the distribution of *Cryptolophocoleasikkimensis* is quite unusual within the genus. The vast majority of taxa principally exhibit a different distribution pattern. The phylogenetic tree shows that *Cryptolophocoleasikkimensis* forms a sister branch to all other taxa widespread in Southeast Asia (widely irrigated to Melanesia) and one pantropical species (*Cryptolophocoleaconnata*). This somewhat correlates with the distinctly different distribution and unique morphology of *C.sikkimensis*. The available data are insufficient for determining the morphological evolution pathways and distribution history within the genus. However, *C.sikkimensis* is assumed to belong to an isolated and morphologically specialized branch. The taxon probably had a wide range in the past that is now disjunctively distributed; in fact, the species is ‘locked’ in the mountainous regions from the Sino-Himalaya to Borneo, considering its ecological preferences.

Within Vietnam, the distribution of the species is limited to the peak surroundings of Phan Xi Pan Mt., a refugium containing a number of Sino-Himalayan species (Bakalin et al. 2018). Although we have visited several highest points in North Vietnam over the last five years, we have not found this taxon, despite the ease of recognition of this species in the field and its large size. On the one hand, this indicates the rarity of the taxon in Vietnam; on the other hand, it confirms the disjunctive relict range of the species.

### ﻿Geographical patterns in the genus

*Cryptolophocolea* includes 32 species (including the newly transferred *C.sikkimensis*), of which the species status is questionable for eight (one star in the World Liverwort Checklist, Söderström et al. 2016). The highest taxonomic diversity is found in Australasia and South America. Africa, the tropical zone of Asia, and other regions contain fewer species. In general, based on the distribution data available at https://www.catalogueoflife.org/ (last accessed 12/15/2021), the distribution is as follows:

Australasia and New Zealand contain 10 species, including four restricted to New Zealand and adjacent islands (*Cryptolophocoleaaculeata* (Mitt.) L. Söderstr., *C.helmsiana* (Steph.) L. Söderstr., *C.spinifera* (Hook.f. & Taylor) L. Söderstr., *C.tuberculata* (J.J. Engel) L. Söderstr.), one taxon restricted to Tasmania (*C.connatifolia* (J.J. Engel) L. Söderstr.), three restricted to Southeast Australia and New Zealand (*C.trialata* (Gottsche) L. Söderstr., *C.subopposita* (J.J. Engel) L. Söderstr., *C.pallida* (Mitt.) L. Söderstr.), and two restricted to Tasmania, New Zealand, Antipodean Islands and some other small adjacent islands (*C.leucophylla* (Hook.f. & Taylor) L. Söderstr., *C.mitteniana* (Colenso) L. Söderstr.).

*Cryptolophocoleachiloscyphoidea* (Lindenb.) L. Söderstr. & Crand.-Stotl. is broadly distributed in Australasia, South America, and the subantarctic islands (and also recorded in India, but that record may be doubted for phytogeographic reasons). South America contains four taxa (in addition to the one mentioned above): *C.fleischeri* (Steph.) L. Söderstr. (also in Mexico), *C.proteus* (Herzog) L. Söderstr., *C.pycnophylla* (Spruce) L. Söderstr., *C.tricorata* (Hässel) Crand.-Stotl. & Stotler.

*Cryptolophocoleaconnata* (Sw.) L. Söderstr. & Váňa is broadly distributed from Africa to Malesia, Australasia, the Neotropics and Polynesia (Hawaii). Africa and South America contain two species that extend beyond this large region: *C.martiana* (Nees) L. Söderstr. (also in the southern part of the U.S.A.) and *C.pallidovirens* (Hook.f. & Taylor) L. Söderstr. (also circumsubantarctic by subantarctic island). Africa has a restricted distribution of *C.lilliena* (Steph.) L. Söderstr. (Kenya only) and *C.regularis* (Steph.) L. Söderstr. (Madagascar, Réunion, and Mauritius). South Asia contains *C.fleischeri* (Steph.) L. Söderstr. (Sri Lanka only). *C.compacta* (Mitt.) L. Söderstr. is strictly found in temperate East Asia (East China, Korea, Japan, also a questionable record from Thailand).

The large region stretching from Southeast Asia (Indochina) to Melanesia contains eight species, with three species distributed across this large area: *C.ciliolata* (Nees) L. Söderstr., Crand.-Stotl., Stotler & Váňa (also in southeast China (Hainan, Taiwan), Sri Lanka in south Asia and Hawaii in Polynesia), *C.costata* (Nees) L. Söderstr. (also in Taiwan) and *C.edentata* (J.J. Engel) L. Söderstr. (also in Taiwan). Melanesia has a restricted distribution of *C.explanata* (Mitt.) Váňa & Crand.-Stotl. (New Caledonia and Samoa). Malesia and Melanesia have a restricted distribution of *C.levieri* (Schiffn.) L. Söderstr. Malesia additionally contains three species: *C.massalongoana* (Schiffn.) L. Söderstr., *C.stephanii* (Schiffn.) L. Söderstr. (Java only), *C.thermarum* (Schiffn.) L. Söderstr. (Java only). Finally, Polynesia contains *C.whittieriana* (Inoue & H.A.Mill.) L. Söderstr. (Caroline Island only).

Therefore, the highest taxonomic diversity is found in New Zealand and adjacent islands (and, to some extent, Tasmania); a less prominent taxonomic ‘peak’ can be found in the southern part of South America, and the third-most taxonomically diverse area is Malesia to Melanesia. The Indochina Peninsula (north Thailand only) contains four species. Northwards of Indochina, the distinctly East Asian *Cryptolophocoleacompacta* and predominantly Paleotropical *C.ciliolata* (reaching Yunnan Province in China) are found. None of the species listed are referred to as Sino-Himalayan floral elements. Therefore, *C.sikkimensis* is the first known species whose area core is distinctly Sino-Himalayan.

## ﻿Conclusions

*Cryptolophocoleasikkimensis* possesses generally narrowly pointed ovate leaves that are unique in the genus. Its phylogenetic affinity could not be clearly identified without molecular genetic investigations. In the present study, such bright and easily noted leaf features were the only possible variants of morphological pathways that occurred in the genus, whereas the underleaves (widely connate with the leaves), and biseriate antheridium stalk show much stronger taxonomic value. The species’ atavistic traits are generally typically evidenced by depauperate plants with bidentate leaves and underleaves. The unique morphology of *C.sikkimensis* is associated with its unique distribution – the species has the only predominantly Sino-Himalayan distribution in the genus.

## Supplementary Material

XML Treatment for Cryptolophocolea sikkimensis

## References

[B1] Bakalin VA Nguyen VS Borovichev EA (2018) New liverwort records for Vietnam. Journal of Bryology 40(1): 68–73. 10.1080/03736687.2017.1393140

[B2] Bakalin V Maltseva Y Vilnet A Choi SS (2021) The transfer of *Tritomariakoreana* to *Lophozia* has led to recircumscription of the genus and shown convergence in Lophoziaceae (Hepaticae). Phytotaxa 512(1): 041–056. 10.11646/phytotaxa.512.1.3

[B3] Borchsenius F (2009) FastGap 1.2. http://www.aubot.dk/FastGap_home.htm [accessed 20 March 2022]

[B4] Chen YS Deng T Zhou Z Sun H (2018) Is the East Asian flora ancient or not? National Science Review 5(6): 142–154. 10.1093/nsr/nwx156

[B5] Cooper ED Shaw AJ Shaw B Henwood MJ Heslewood MM Brown EA (2011) A multi-locus molecular phylogeny of the Lepidoziaceae: Laying the foundations for a stable classiﬁcation. Molecular Phylogenetics and Evolution 59(2): 489–509. 10.1016/j.ympev.2011.02.006 21316477

[B6] Dimon RJ Váňa J Schäfer-Verwimp A Heinrichs J Renner MAM (2018) *Conoscyphus* belongs to *Acrobolbaceae* (Jungermanniineae) not *Lophocoleaceae* (Lophocoleineae). Australian Systematic Botany 31(3): 209–218. 10.1071/SB17041

[B7] Engel JJ Schuster RM (1985) [“1984”)] An overview and evaluation of the genera of Geocalycaceae subfamily Lophocoleoideae (Hepaticae). Nova Hedwigia 39: 385–463.

[B8] Feldberg K Váňa J Krusche J Kretschmann J Patzak SDF Pérez-Escobar OA Rudolf NR Seefelder N Schäfer-Verwimp A David GL Schneider H Heinrichs J (2016) A phylogeny of Cephaloziaceae (Jungermanniopsida) based on nuclear and chloroplast DNA markers. Organisms, Diversity & Evolution 16(4): 727–742. 10.1007/s13127-016-0284-4

[B9] Hall TA (1999) BioEdit: A user-friendly biological sequence alignment editor and analysis program for Windows 95/98/NT. Nucleic Acids Symposium Series 41: 95–98.

[B10] Hentschel J Wilson R Burghardt M Zündorf H-J Schneider H Heinrichs J (2006a) Reinstatement of Lophocoleaceae (Jungermanniopsida) based on chloroplast gene *rbc*L data: Exploring the importance of female involucres for the systematics of Jungermanniales. Plant Systematics and Evolution 258(3–4): 211–226. 10.1007/s00606-006-0408-y

[B11] Hentschel J Zündorf H-J Hellwig FH Schäfer-Verwimp A Heinrichs J (2006b) Taxonomic studies in *Chiloscyphus* Corda (Jungermanniales: Lophocoleaceae) based on *nr*ITS sequences and morphology. Plant Systematics and Evolution 258: 211–226. 10.1007/s00606-006-0408-y

[B12] Hentschel J Feldberg K Zündorf H-J Hellwig FH Schneider H Heinrichs J (2007) The systematic position of *Pachyglossa* and *Clasmatocolea* (Jungermanniopsida: Lophocoleaceae) inferred from nrDNA ITS sequences and morphology. Taxon 56(4): 1136–1142. 10.2307/25065908

[B13] Herzog T (1939) Zwei Bryophytensammulgen aus dem Sikkim Himalaya. Annales Bryologici 12: 71–97.

[B14] Herzog T Grolle R (1958) Was ist *Pachyglossa*? Revue Bryologique et Lichénologique 27(3–4): 147–165.

[B15] Katoh K Standley DM (2013) MAFFT multiple sequence alignment software version 7: Improvements in performance and usability. Molecular Biology and Evolution 30(4): 772–780. 10.1093/molbev/mst010 23329690 PMC3603318

[B16] Kitagawa N (1974) A study of *Lophocoleasikkimensis*. Bulletin of Nara University of Education 23: 31–40.

[B17] Kumar S Stecher G Li M Knyaz C Tamura K (2018) MEGA X: Molecular Evolutionary Genetics Analysis across computing platforms. Molecular Biology and Evolution 35(6): 1547–1549. 10.1093/molbev/msy096 29722887 PMC5967553

[B18] Long D Grolle R (1990) Hepaticae of Bhutan. II. The Journal of the Hattori Botanical Laboratory 68: 381–440.

[B19] Milyutina IA Goryunov DV Ignatov MS Ignatova EA Troitsky AV (2010) The phylogeny of *Schistidium* (Bryophyta, Grimmiaceae) based on the primary and secondary structure of nuclear rDNA internal transcribed spacers. Molecular Biology 44(6): 883–897. 10.1134/S0026893310060051 21290822

[B20] Nguyen L-T Schmidt HA von Haeseler A Minh BQ (2015) IQ-TREE: A fast and effective stochastic algorithm for estimating maximum likelihood phylogenies. Molecular Biology and Evolution 32(1): 268–274. 10.1093/molbev/msu300 25371430 PMC4271533

[B21] Pacak A Szweykowska-Kulinska Z (2000) Molecular data concerning alloploid character and the origin of chloroplast and mitochondrial genomes in the liverwort species *Pelliaborealis*. Journal of Plant Biotechnology 2: 101–108.

[B22] Patzak SDF Renner MAM Schäfer-Verwimp A Feldberg K Heslewood MM Peralta DF Heinrichs J (2016) A phylogeny of Lophocoleaceae-Plagiochilaceae-Brevianthaceae and a revised classification of Plagiochilaceae. Organisms, Diversity & Evolution 16(3): 481–495. 10.1007/s13127-015-0258-y

[B23] Piippo S (1990) Annotated catalogue of Chinese Hepaticae and Anthocerotae. The Journal of the Hattori Botanical Laboratory 68: 1–192.

[B24] Piippo S He XL Koponen T Redfearn Jr PJ Li JX (1998) Hepaticae from Yunnan, China, with a checklist Of Yunnan Hepaticae and and Anthocerotae. The Journal of the Hattori Botanical Laboratory 84: 135–158.

[B25] Ronquist F Teslenko M van der Mark P Ayres DL Darling S Höhna S Larget B Liu L Suchard MA Huelsenbeck JP (2012) MrBayes 3.2: Efficient Bayesian phylogenetic inference and model choice across a large model space. Systematic Biology 61(3): 539–542. 10.1093/sysbio/sys029 22357727 PMC3329765

[B26] Söderström L Crandall-Stotler B Stotler RE Váňa J Hagborg A von Konrat M (2013) Notes on Early Land plants Today. 36. Generic treatment of Lophocoleaceae (Marchantiophyta). Phytotaxa 97(2): 36–43. 10.11646/phytotaxa.97.2.3

[B27] Söderström L Hagborg A von Konrat M Bartholomew-Began S Bell D Briscoe L Brown E Cargill DC Costa DP Crandall-Stotler BJ Cooper ED Dauphin G Engel JJ Feldberg K Glenny D Gradstein SR He X Heinrichs J Hentschel J Ilkiu-Borges AL Katagiri T Konstantinova NA Larrain J Long DG Nebel M Pócs T Felisa Puche F Reiner-Drehwald E Renner MAM Sass-Gyarmati A Schäfer-Verwimp A Moragues JGS Stotler RE Sukkharak P Thiers BM Uribe J Váňa J Villarreal JC Wigginton M Zhang L Zhu R-L (2016) World checklist of hornworts and liverworts. PhytoKeys 59: 1–828. 10.3897/phytokeys.59.6261 PMC4758082 26929706

[B28] Stephani F (1922) Species hepaticarum 6. George & Cie, Genève & Bale, 241–368. 10.5962/bhl.title.95494

[B29] Taberlet P Gielly L Pautou G Bouvet J (1991) Universal primers for amplification of three non-coding regions of chloroplast DNA. Plant Molecular Biology 17(5): 1105–1109. 10.1007/BF00037152 1932684

[B30] Wang J Lai MJ Zhu RL (2011) Liverworts and hornworts of Taiwan: an updated checklist and floristic accounts. Annales Botanici Fennici 48: 369–395.

